# Recent advances in the pathogenesis and prevention strategies of dental calculus

**DOI:** 10.1038/s41522-024-00529-1

**Published:** 2024-07-13

**Authors:** Yu Wei, Gao-peng Dang, Zhao-yang Ren, Mei-chen Wan, Chen-yu Wang, Hong-bo Li, Tong Zhang, Franklin R. Tay, Li-na Niu

**Affiliations:** 1 State Key Laboratory of Oral & Maxillofacial Reconstruction and Regeneration, National Clinical Research Center for Oral Diseases, Shaanxi Key Laboratory of Stomatology, Department of Prosthodontics, School of Stomatology, The Fourth Military Medical University, Xi’an, Shaanxi China; 2 Department of Stomatology, the First Medical Center, Chinese PLA General Hospital, Beijing, China; 3 The Dental College of Georgia, Augusta University, Augusta, GA USA

**Keywords:** Bacteria, Biofilms

## Abstract

Dental calculus severely affects the oral health of humans and animal pets. Calculus deposition affects the gingival appearance and causes inflammation. Failure to remove dental calculus from the dentition results in oral diseases such as periodontitis. Apart from adversely affecting oral health, some systemic diseases are closely related to dental calculus deposition. Hence, identifying the mechanisms of dental calculus formation helps protect oral and systemic health. A plethora of biological and physicochemical factors contribute to the physiological equilibrium in the oral cavity. Bacteria are an important part of the equation. Calculus formation commences when the bacterial equilibrium is broken. Bacteria accumulate locally and form biofilms on the tooth surface. The bacteria promote increases in local calcium and phosphorus concentrations, which triggers biomineralization and the development of dental calculus. Current treatments only help to relieve the symptoms caused by calculus deposition. These symptoms are prone to relapse if calculus removal is not under control. There is a need for a treatment regime that combines short-term and long-term goals in addressing calculus formation. The present review introduces the mechanisms of dental calculus formation, influencing factors, and the relationship between dental calculus and several systemic diseases. This is followed by the presentation of a conceptual solution for improving existing treatment strategies and minimizing recurrence.

## Introduction

Dental calculusis a common health problem in the human body. It has a high rate of occurrence (~90%) among the general population^1,2^. Recent studies reported that dental calculus is also associated with digestive, neurological, and cardiovascular diseases (Table 1). The quality of life of the involved subjects is seriously threatened if the secondary harm caused by dental calculus is not appropriately addressed.

**Table 1:** Secondary diseases and their pathogenic factors due to dental calculus

Body Part	Disease	Pathogenic Factor	Refs.
Oral cavity	Gingivitis	Bacterial biofilm	^75,76^
	Periodontitis	Bacterial biofilm; the NLRP3 inflammasome	^77^
	Oral submucous fibrosis	Copper in dental calculus	^78^
	Oral squamous cell carcinoma	Cadmium in dental calculus	^79^
Digestive system	Colorectal cancer	*P. gingivalis*; the NLRP3 inflammasome	^80^
	Esophageal cancer	*P. gingivalis; Tannerella forsythia*	^81,82^
	Inflammatory bowel disease	Ectopic colonization of oral pathogens; the recruitment of macrophages and T cells	^83,84^
Nervous system	Blood-brain barrier damage	*P. gingivalis*	^85^
	Alzheimer’s disease	Gingipains	^86^
	Cerebral abscesses	Bacterial infection	^87^
	Stroke	*P. gingivalis*	^88,89^
Cardiovascular system	Combined hypertension	Dysbiosis of oral microbiota; damage to vascular epithelium; immune responses	^90,91^
	Angina pectoris	-	^92^
	Heart infarction	Bacteremia; endotoxemia	^93^
	Atherosclerosis	*P.gingivalis*	^94^

Dental calculus is difficult to remove. Its rough surface provides good conditions for bacteria adhesion and growth. As a cradle for bacterial colonization and reproduction, dental calculus is most likely to accumulate along the gingival margins. Bacteria present in dental calculus continuously irritate periodontal tissue. Bone resorption, periodontal pocket formation, periodontal pus overflow, root exposure, and tooth loss inevitably occur during the advanced stages of periodontitis^3^.

**Fig. 1:**
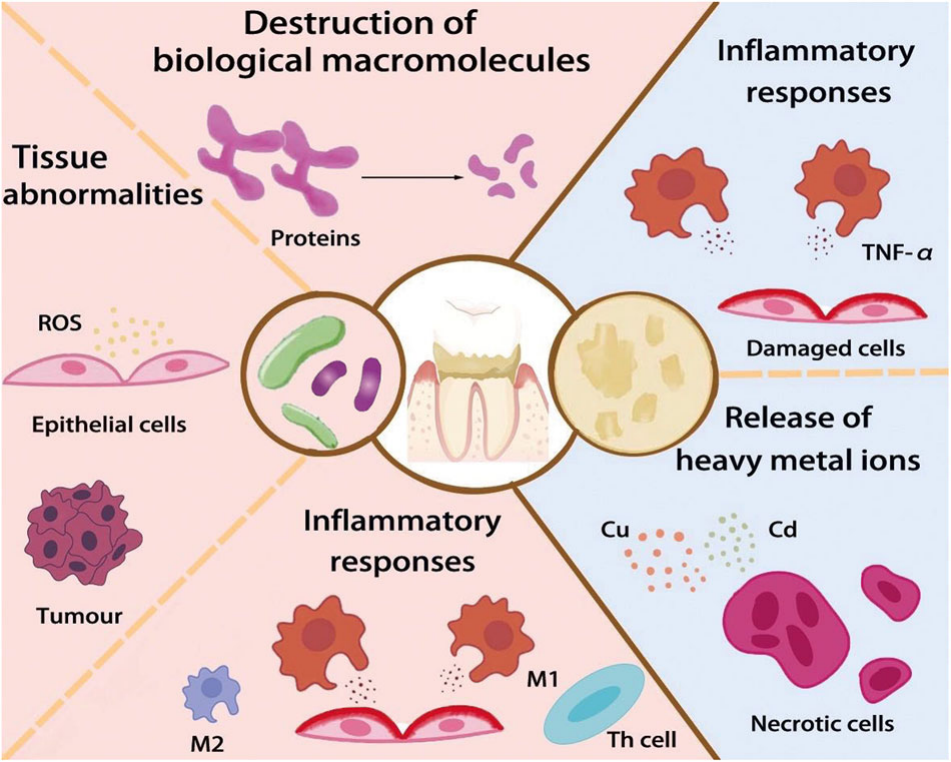
Pathogenesis of dental calculus involves bacteria and matrix mineralization. Bacteria induce destruction of biological macromolecules, tissue abnormalities, and inflammatory responses. Mineralized matrix releases heavy metal ions and causes inflammatory responses. (ROS reactive oxygen species, M1 classically activated macrophages, M2 alternatively activated macrophages, Th cells T helper cells, TNF-a tumor necrosis factor-a) (The figure does not contain any third-party material, the figure and each of the elements in the figure were created by the authors).

The present review first introduces the mechanisms of dental calculus formation. Multiple factors contribute to the formation of the calculus. These factors may be divided into bacterial factors, immune factors, and host factors. These three factors interact with each other to promote calculus formation (Fig. 1). Novel strategies for the prevention of dental calculus have also been developed over the past few years. These strategies include laser technology, nanotechnology, and the application of biological agents. These preventive and treatment strategies will be discussed, from principles to application, to provide insight for future research.

## Mechanisms of dental calculus formation

Dental calculus consists of inorganic components and an organic matrix derived from saliva, gingival crevicular fluid, and bacterial products^4^. Bacteria are critical for the formation of dental calculus. They form biofilms on the surface of natural teeth and dentures. The biofilms undergo mineralization and become dental calculus^5^. This section introduces the mechanisms of dental calculus formation by focusing on the changes in bacteria.

### Bacterial factors

Dental calculus forms through the mineralization of dental plaque, and can persist on ancient skeletal remains for thousands of years. These remains are capable of preserving ancient biomolecules, including intact DNA and proteins^6^. Consequently, researchers have utilized ancient dental calculus to examine the composition and function of oral microbial communities. Key oral pathogens have been identified from ancient dental calculus^7^. Furthermore, analyses of ancient dental calculus suggest that changes in bacterial diversity correlate with dietary shifts and variations in social environments^8^.

#### Formation and functions of bacterial biofilm and matrix

**Fig. 2:**
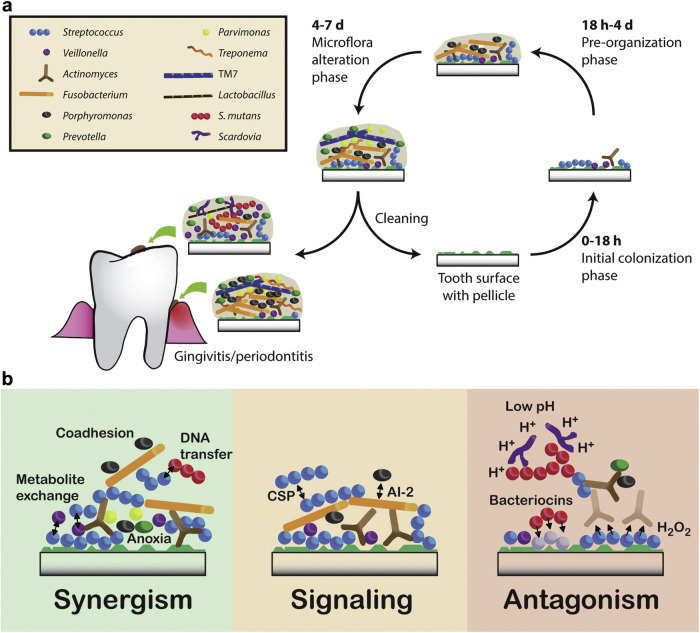
Formation and development of plaque biofilms. **a** On natural tooth surfaces, initial colonization takes place over 0–18 h and, at this point, the biofilm is dominated by pioneer colonizers that are adapted to adhere to saliva pellicle constituents and to tolerate the presence of oxygen. If left unperturbed, the biofilm gradually matures without dramatic shifts in the microbial composition between 18 h and 4 days. From 4 to 7 days, obligate anaerobes increase at the gum margins. **b** Interactions between microorganisms that may drive the community assembly of oral biofilms^71^. **a** and **b** are reproduced with permission from the publisher.

Bacterial biofilm consists of bacteria that are surrounded by a polymeric matrix produced by the bacteria^9^. The process of bacterial biofilm formation has been well investigated. Bacteria first attach reversibly to teeth through van der Waals forces and hydrophobic interactions. They use their pili to irreversibly attach to tooth surfaces, and form colonies to stabilize their attachment^10^. During the process, a self-produced matrix of extracellular polymeric substance (EPS) acts as a hard shell to protect the bacteria from external pressure^11^. The EPS has additional functions such as mediation of bacteria migration and provision of signaling targets. Accumulation of small bacterial clusters and bacterial layers subsequently results in the formation of plaque biofilm^12^. Channels and pores within the biofilm help the circulation of nutrition between the internal biofilm milieu and the exterior environment^13^. Bacteria present in dental plaque are divided into early colonizers and late colonizers. Early colonizers are typically facultative anaerobes and saccharolytic species that thrive on salivary mucins and other glycoproteins^3^. Late colonizers are usually proteolytic obligate anaerobes. The “bridge organism” between early and late colonizers is *Fusobacterium nucleatum*. This bacterium is capable of co-aggregating with other bacteria species^14^. Mature biofilms provide a platform for mineralization^15^. Among the plethora of bacteria present in plaque biofilms, *Streptococcus sanguinis* is the primary colonizer. *Streptococcus mutans* and *Streptococcus gordonii* co-aggregate over the primary colonizer via molecular interactions. The variety of bacterial species and interaction between distinct layers results in the formation of stable plaque biofilms^16^ (Fig. 2). Changes within the bacteria also play an important role in mature biofilms. As a second messenger, intracellular cyclic di-guanosine monophosphate promotes surface attachment of biofilms. Reduction in intracellular cyclic di-guanosine monophosphate causes biofilm dispersion^17^.

**Fig. 3:**
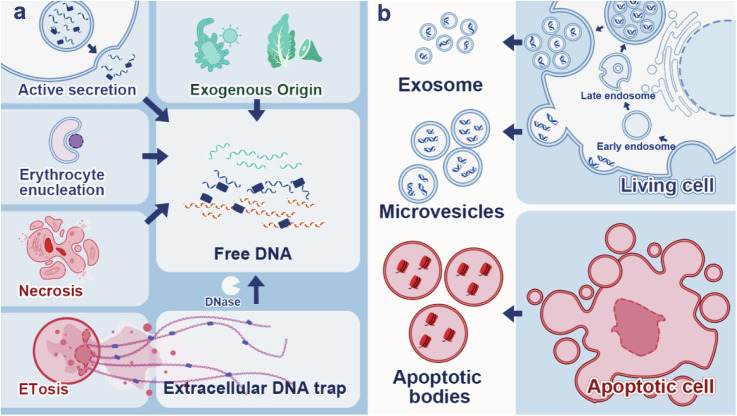
The origin of exDNA. **a** Free DNA is derived from multiple sources. These molecules are produced mainly by endogenous pathways, including active secretion, erythrocyte nucleation, necrosis, and degradation of ETs from neutrophils. **b** Three types of extracellular vesicles carry exDNA^22^. Figure reproduced with permission from the publisher.

The composition of the extracellular matrix also affects the status of plaque biofilms^18^. Compared with healthy teeth samples, teeth with dental calculus have more bacterial virulence proteins, which worsen the periodontal microenvironments. Glucosyltransferases produced by bacteria are released to the dental calculus matrix by exocytosis^19^. These enzymes catalyze the production of exopolysaccharides and promote bacteria adhesion to the surface of early biofilms^20^. Matrix macromolecules such as extracellular DNA (exDNA) are characteristic of mature biofilm in periodontitis. Extracellular DNA exists in the matrix in two forms: free DNA and extracellular DNA traps(ETs) (Fig. 3). Irrespective of the origin of exDNA, these molecules play a crucial role in initiating biofilm formation and sustaining the biofilm’s three-dimensional structure^21,22^. The adhesion of *S. mutans* to hydrophobic surfaces and the viscoelasticity of biofilms are enhanced by exDNA. Under nutrient-limiting conditions, bacteria release exDNA enzymes that utilize exDNA as a surrogate source of nutrition^23^. Besides, Quorum sensing among biofilm bacteria, cyclic diguanylate, and small non-coding RNAs within the matrix regulatory network is involved in the regulation of biofilm formation^24^.

Biofilm formation on dental implants and teeth follows a similar pattern. However, there are a few differences due to the implant materials used. These materials have a lower capacity for albumin absorption compared with natural teeth. This property results in reduced biofilm formation around implants. The process of biofilm formation begins on the implant surface ~30 min after exposure to the oral environment. In contrast, biofilms around natural teeth form within minutes^25^. Initially, implants are primarily colonized by gram-positive cocci, rods, and actinomyces species, which attach to salivary agglutinins and proline-rich proteins. These early colonizers pave the way for subsequent attachment by late colonizers such as *Aggregatibacter actinomycetemcomitans*, *Porphyromonas gingivalis*, and *Fusobacterium nucleatum*. The stepwise colonization by bacteria facilitates the development of biofilm and escalates its pathogenicity over time^26^. In addition, microgaps ranging from 2.5–60 μm in the implant-abutment interface impair the seal, allowing bacteria to infiltrate and form biofilms within the implant’s inner threads^27^. Biofilms contribute to the formation of dental calculus, which in turn deteriorates the surface of the implant and increases its roughness. This roughness fosters further bacterial re-colonization, hindering the reattachment and proliferation of host cells^28^.

#### Mechanism of dental calculus biofilm mineralization

Mature plaque biofilms formed on the surfaces of teeth and dentures provide platforms for mineralization. Plaque bacteria are engaged in different metabolic processes that interact with inorganic minerals to produce mineral precipitates in dental calculus. The most common minerals deposited by bacteria are calcium carbonate and calcium phosphate^29^. The most frequent calcium phosphate crystalline phases found in dental calculus are hydroxyapatite, octacalcium phosphate, and whitlockite^30^. However, the local concentrations of calcium and phosphate ions are not high enough to cause spontaneous precipitation^31^. Therefore, bacteria play an important role in mineral deposition. It is necessary to understand the mineralization mechanism of the interaction between bacteria and minerals.

Bacteria release different enzymes during the mineralization process. Recently, the release of alkaline phosphatase (ALP) was reported to be associated with periodontal inflammatory responses^32^. ALP has also been identified as a hallmark enzyme in periodontitis. Microorganisms in the dental biofilm induce the death of epithelial cells. Destruction of the epithelial barrier induces infected epithelial cells and immune cells to produce pro-inflammatory cytokines and chemokines, such as IL-1β, IL-6, and IL-8^33^. The pro-inflammatory cytokines recruit inflammatory cells and initiate the subsequent inflammatory response. The ALP is released by bacteria^34^. Bacterial ALPs are found extracellularly, bonding on the surface of the bacteria. Alkaline phosphatase has the ability to dephosphorylate teichoic acid, a molecule that plays a key role in bacterial colonization on teeth. With the release of phosphate ions, calcium ions may become supersaturated^35^. It is also reported that ALP regulated biofilm formation by promoting aerobic pathways^35^. This shows the importance of ALP in the mineralization of biofilm.

In addition, positively-charged polycationic compounds also induce dental calculus mineralizati^on^^36^. For instance, *streptococcal* bacterial protein is rich in positively-charged amino acids. It acts as a substrate for the nucleation and growth of inorganic mineral particles^37^. Environmental changes also affect dental calculus mineralization. Bacteria produce enzymes that influence the environment and regulate calcium and phosphorus content^38^. Ureolytic bacteria hydrolyze salivary and dietary urea via the enzyme urease to produce ammonia and carbon dioxide, increasing the pH value. Thus, calcium phosphate saturation increases in plaque fluid and accelerates the formation of dental calculus^17^.

#### Effects of physicochemical factors on bacteria

**Fig. 4:**
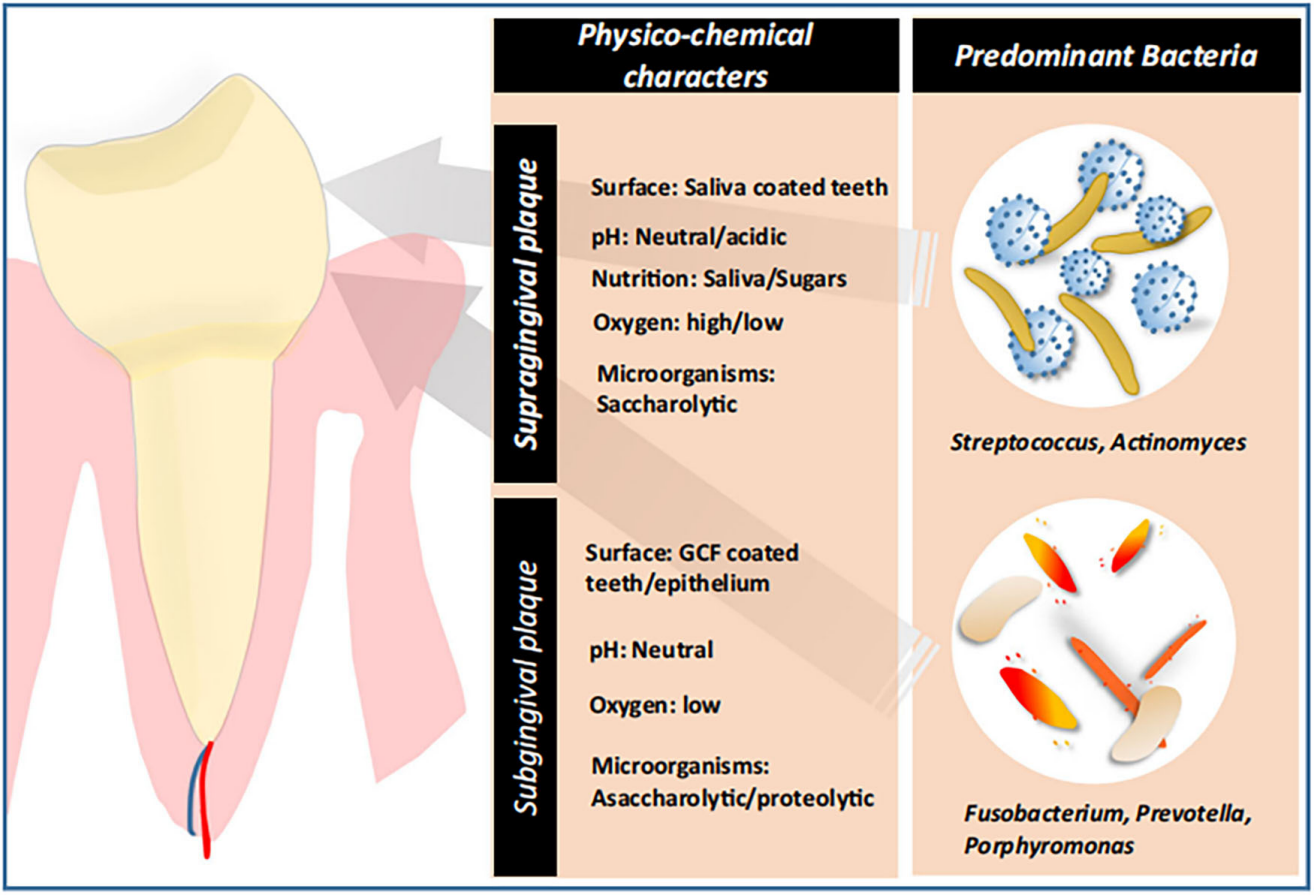
Physical and chemical factors present in supragingival or subgingival plaque. The types of bacteria located in the supragingival and subgingival plaques are different. The physicochemical factors affecting the bacteria in the two sites are also different^72^. This schematic is reproduced with permission from the publisher.

The relationship between the oral microbiota and the oral physicochemical environment changes dynamically. This relationship may evolve from a healthy symbiotic relationship to pathological dysbiosis^39^. Studying the influence of oral physicochemical factors on bacterial status can help elucidate the mechanisms of calculus formation. In addition, changes in physical and chemical factors may also cause changes in supragingival or subgingival plaque microflora (Fig. 4).

### Immune factors

**Fig. 5:**
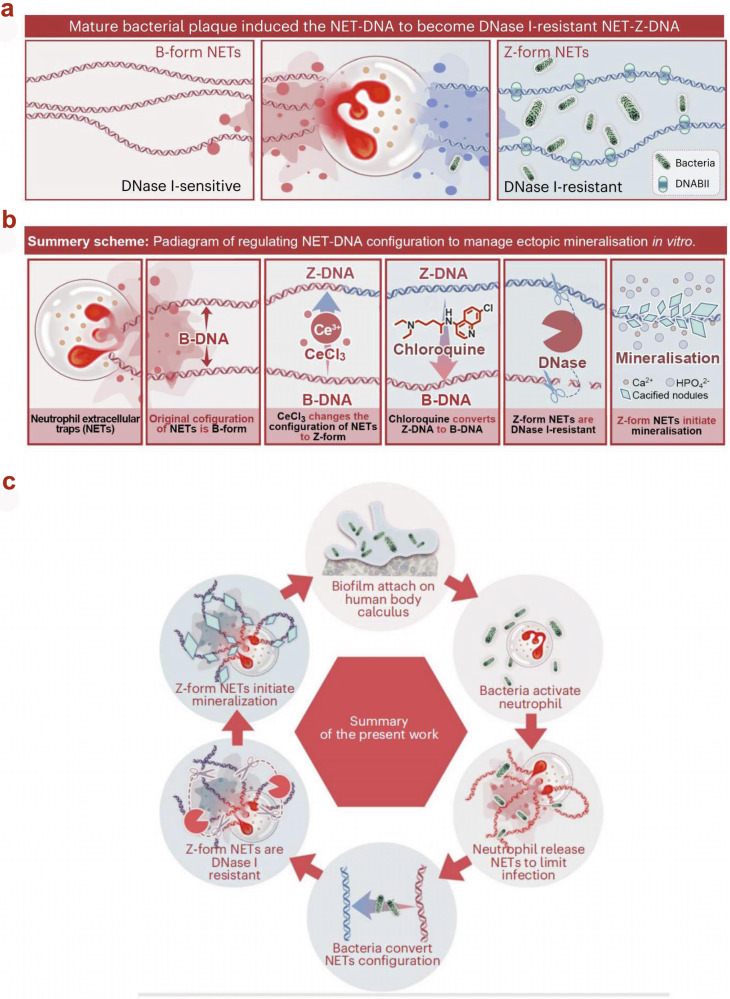
Formation of dental calculus in the presence of NETs. **a** Bacterial biofilms trigger the transformation of NET-DNA into DNase-I-resistant NET-Z-DNA. **b** NET-DNA plays a role in regulating ectopic mineralization in vitro. **c** The mineralization mechanism of dental calculus involving NETs^46^. **a**, **b**, and **c** are reproduced with permission from the publisher.

Periodontal crevices and pockets are filled with gingival crevicular fluid. The latter is rich in immune cells. Under the stimulus of a biofilm, neutrophils^40^, and macrophages^41^ are recruited to the pocket site and activated. Neutrophil extracellular traps (NETs), secreted by neutrophils, play a dual role in both protecting the host from infections and facilitating the mineralization of dental calculus. These NETs are capable of capturing pathogens, thereby limiting infection^42^. There is substantial evidence suggesting that NETs are instrumental in the formation of gallstones, salivary stones, and monosodium urate crystals^43–45^. A recent study revealed that NETs-DNA acts as nucleation sites for pathological mineralization of dental calculus. The study demonstrated that bacteria within the mature biofilms secrete histone-like proteins that alter the chiral structure of NETs-DNA from the B-form to the Z-form. This transformation reduces the susceptibility of NETs to degradation by deoxyribonuclease I, thereby enhancing their ability to induce mineralization^46^ (Fig. 5). Bacteria and mineralized crystallites further recruit and polarize macrophages into classically-activated subtypes^47^. The activated macrophages release exosome-like calcifying extracellular vesicles. These vesicles are rich in calcium ions and are capable of mineralization. During the formation of calculus, neutrophils act more promptly than macrophages^48^. Hence, it is important to reduce the contribution of neutrophils to mineralization. Targeted inhibition of extracellular nuclease released by bacteria may enhance the efficiency of neutrophils and delay the mineralization of bacterial biofilms.

### Host factors

Lifestyles have significant effects on calcium phosphate saturation and bacterial colonization. Tobacco smoking induces higher salivary calcium contents which increase the formation of calculus^49–51^. A carbohydrate-dominated diet may induce dental calculus by promoting bacterial colonization on the plaque53. Sucrose is the most widely consumed dietary carbohydrate. This sugar modifies the local microenvironment, and is closely associated with increased dysbiosis in oral biofilms^52^. Replacing refined carbohydrates with foods high in fat and vitamin D could lessen bacterial adhesion to teeth^53^. However, it is necessary to ascertain whether individuals aiming to mitigate dental calculus find such dietary substitutions acceptable.

## Novel prevention and treatment strategies for dental calculus

Conventional methods of dental calculus prevention and treatment are brushing, scaling, and root planning (SRP). Improving oral hygiene by brushing or using chlorhexidine mouth rinses is the most economical and convenient way to prevent the development of calculus^54^. The use of SRP polishes the tooth surface to prevent the re-deposition of biofilms and dental calculi^55^. Given the main pathogenic factors of periodontitis are dental calculus and plaque that are attached to the root surface, this oral disease may be treated by subgingival scaling. This treatment reduces the depth of periodontal pockets and renders the root surface smooth. The condition that is favorable to facilitate the reattachment of the gingiva to the root surface is created^56^. These changes reduce the chance for bacteria to attach. Apart from physical removal techniques, chemical agents such as pyrophosphates play a crucial role in dental calculus management. Pyrophosphates disrupt bacterial growth by hindering the biosynthesis of the bacterial cell wall, which obstructs lipid transport essential for cell wall integrity^55^. These compounds also lower calcium and phosphorus concentrations in the periodontal microenvironment. Consequently, pyrophosphates contribute to reducing the essential components required for bacterial mineralization^57^. This helps to prevent dental calculus formation.

Innovative treatment modalities for dental calculus have emerged in recent years. These include laser technology, new kinds of toothpaste, and natural plant-derived compounds^58,59^. In addition, vaccines and nanotechnology offer unique perspectives for the control of dental calculus.

Physical intervention is a common approach to preventing and treating dental calculus. Ultrasound relies on cavitation and acoustic microstreaming to achieve cleaning^60^. Shockwaves generated by lasers alter biofilm permeability to enable easier elimination of pathogenic bacteria^61^. Calculus removal with lasers leaves no residual calculi, which is better when compared with conventional manual instruments^62^. However, the use of erbium-doped yttrium/aluminum/garnet laser and erbium-chromium-doped yttrium/scandium/gallium/garnet laser results in rougher root surfaces^56^. A combination of SRP and erbium laser is the solution to solve the problem. It removes residual debris from the root surface with little or no adverse thermal damage on the root surface^63^. Thermal damage, especially increased temperature within the periodontium, is considered a potential hazard to the dental pulp and periodontal tissues^64^. Thus, the advantage of laser technology for calculus removal is relatively limited due to the risk of thermal damage^59^. The effectiveness of the erbium laser and SRP in non-surgical periodontal treatment needs further evaluation.

Damage to patients’ enamel surfaces with manual and ultrasonic scalers has been a problem that plagued clinicians. To address this issue, scientists developed a novel actuator-driven pulsed water jet (ADPJ) system^65^. The system is capable of selectively removing materials based on their hardness. Because of the different hardness of teeth and dental calculus, the ADPJ system removes dental calculus with appropriate jet pressure without damaging the enamel surface of the tooth^66^. The ADPJ system was shown to significantly reduce the amount of dental calculus, and the removal rate was dependent on the applied voltage. No enamel damage was observed on the tooth surface after using the ADPJ system when compared to conventional scalers.

Aragonite (a calcium carbonate mineral phase) can be extracted from animals such as cuttlefish. It is an abrasive with a hardness that facilitates the removal of dental calculus^67^. Scientists tested the effectiveness of aragonite toothpaste derived from squid to remove dental calculus^53^. The aragonite toothpaste had a good effect in removing calculus, preventing calculus formation, and improving gingival health. The aragonite toothpaste dud bit causes any enamel damage^53^.

Vaccines have been anticipated to improve the prevention and treatment of periodontal disease. However, the immunopathological complexity of periodontal disease complicates the development of periodontal vaccines^57^. Studies suggest that successful periodontal vaccines may require mucosal delivery and polyvalent approaches. This strategy requires clinical trials for validation of the treatment outcomes^57^.

**Fig. 6:**
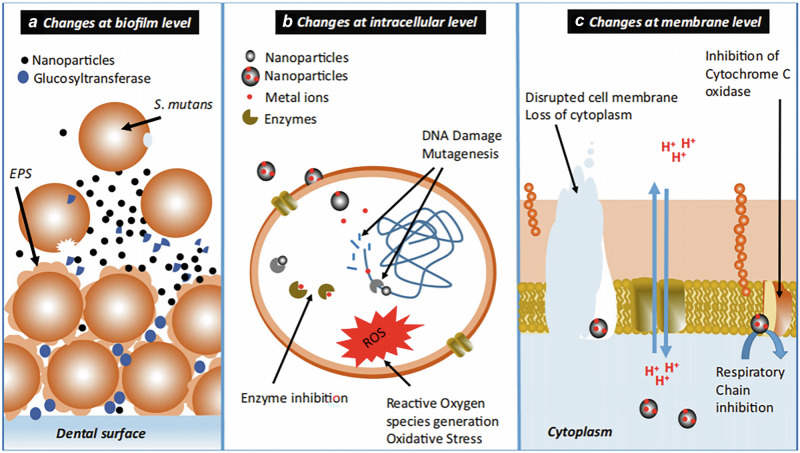
Mechanisms of nanoparticle-mediated antimicrobial and anti-biofilm activities against oral bacteria. **a** The inhibition of glucosyl transferase by nanoparticles results in reduced exopolysaccharide production and biofilm formation. **b** Nanoparticle-mediated biochemical changes occur at the cellular level. **c** The changes take place in the membranes of individual cells^72^. **a**, **b**, and **c** are reproduced with permission from the publisher.

Other scientists utilized nanoparticles and biological agents to prevent the formation of bacterial biofilms. Metal and metal oxide nanoparticles have broad application prospects due to their small size, large specific surface area, and antibacterial properties^68^. The incorporation of silver and gold nanoparticles in toothbrushes enhances the mechanical control of dental plaque^69^. Platinum nanoparticles have many outstanding features. They provide powerful mechanical control and have the potential to degrade proteins and periodontal disease-associated inflammatory factors, such as lipopolysaccharides^70^. The specific mechanism of nanoparticles on bacteria is shown in Fig. 6.

**Fig. 7:**
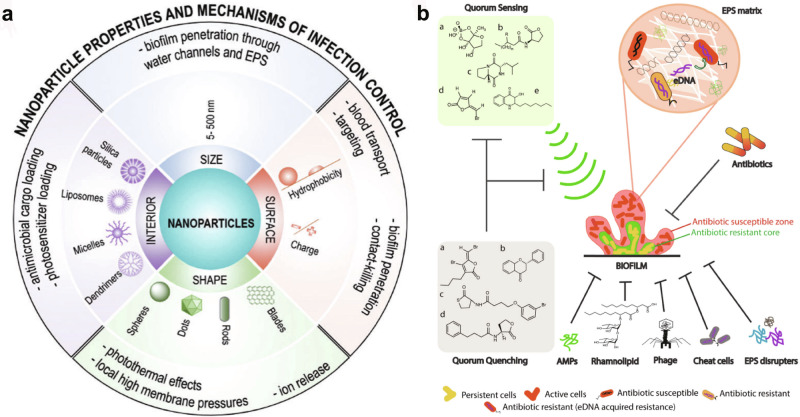
Nanoparticle-mediated antimicrobial and anti-biofilm activities against oral bacteria. **a** Size and shape, surface, and interior properties of nanoparticles are important for their use in biofilm-infection control^73^. **b** Effect of antibiotics on biofilms. Other strategies employed for the treatment of biofilms include the use of antimicrobial peptides (AMP), bio-surfactants (rhamnolipid), phage particles, cheat cells, and different EPS-disrupting enzymes^74^. **a** and **b** are reproduced with permission from the publisher.

Factors such as the size of the nanoparticles can also affect the efficacy of calculus removal. Other novel methods for preventing calculus formation are in their infancy of development (Fig. 7).

Active patient participation and maintenance of good oral hygiene are essential to prevent plaque and biofilm formation. The development of mouthwash or toothpaste may be a promising avenue to facilitate the prevention of calculus formation. These products exhibit low toxicity, high biosafety, and exceptional efficacy. By continuously exploring novel treatment modalities, the incidence of dental calculus will be greatly reduced. This also reduces the potential harm of dental calculus on systemic diseases.

## Outlook

Periodontal diseases caused by dental calculus affect both oral and systemic health. The present review systematically describes the mechanisms of calculus formation in the human oral environment. Considerations need to be given to both bacterial and environmental factors that cause calculus deposition on tooth surfaces. The mechanisms of plaque biofilm formation as well as the alteration of bacterial types during the formation of biofilm and calculus are also highlighted. In addition, the influence of the inflammatory state of the body and the physicochemical state of the saliva on the formation of dental calculus has also been demonstrated. To alleviate clinical problems, new treatment methods for calculus removal have emerged in recent years. However, the interconnection between dental calculus formation and other systemic diseases is not completely understood.

In the future, healthcare workers need to objectively address multiple factors such as age, gender, race, and religious belief. Systematic classification of patients as well as the condition of diseases will be used to design individualized treatment. At the same time, doctors need to raise awareness of the importance of oral hygiene and the early diagnosis of gingival and periodontal problems. To achieve this goal, preventive and curative oral health policies adapted to patients’ diverse living environments need to be planned and implemented, with resources, goals, and priorities identified.

## Data Availability

Data sharing does not apply to this article as no datasets were generated or analyzed during the current study.
